# Trends in Utilization of Emergency Contraceptives in Tanzania: A Retrospective Longitudinal Study From 2018 to 2023

**DOI:** 10.7759/cureus.57649

**Published:** 2024-04-05

**Authors:** Auleria W Kadinde, Raphael Z Sangeda, Lucy Mwenda, Khadija I. Yahya-Malima, Cassian F Masatu, Yonah H Mwalwisi, Adam M Fimbo

**Affiliations:** 1 Pharmaceutical Microbiology, Muhimbili University of Health and Allied Sciences, Dar es Salaam, TZA; 2 Nursing Management, Muhimbili University of Health and Allied Sciences, Dar es Salaam, TZA; 3 Medicines Control, Tanzania Medicines and Medical Devices Authority, Dodoma, TZA

**Keywords:** tanzania, pharmacoepidemiology, levonorgestrel, ethinyloestradiol, p2, emergency contraceptives (ecs)

## Abstract

Introduction

Emergency contraceptives (ECs) are a critical method for preventing unwanted pregnancies following unprotected sexual intercourse. However, Tanzania is experiencing an alarming surge in the misuse of ECs among reproductive-aged females, particularly younger girls and women. Reports of their use as regular contraceptives are a rising concern. Deviations from their intended use in emergencies may not only increase the risk of contraceptive failure but also increase the risk of adverse health events. This study aims to delineate and evaluate the utilization patterns of ECs over six consecutive years using importation data obtained from the Tanzania Medicines and Medical Devices Authority (TMDA).

Materials and methods

We analyzed the EC data collected by TMDA over six consecutive years using a retrospective longitudinal design. Microsoft Power BI (Microsoft^®^ Corp., Redmond, WA) was used to clean, organize, and aggregate the data. IBM SPSS Statistics for Windows, Version 26 (Released 2019; IBM Corp., Armonk, New York, United States) was used to analyze annual trend utilization using linear regression.

Results

We analyzed 114 importation consignments for ECs, identifying 95.6% (109 records) as oral ECs and 4.4% (five records) as intrauterine devices (IUDs) between 2018 and 2023. This data revealed a significant increase in the volume of EC imports, with its contribution increasing from 1.9% in 2018 to 60.1% in 2023. This highlights the marked increase in EC consumption in Tanzania. In 2023, the defined daily dose per 1000 inhabitants per year (DID) peaked at 3.917826, indicating an unprecedented increase of 4,983.06% compared to the lowest DID observed in 2019 at 0.0873552. The year 2023 alone accounted for 41.63% of the total DID (9.43) over the entire study period. In 2019 and 2020, there was a decrease in EC consumption, followed by a rapid increase from 2021 to 2023. The reduction in EC consumption from 2019 to 2020 was 36.9% compared to that between 2021 and 2022.

Conclusions

The significant rise in EC importation and utilization in Tanzania between 2018 and 2023, marked by fluctuating consumption trends and a notable surge, highlights the urgent need for targeted educational and policy intervention. This will guide the rational and informed use of ECs, ensuring access aligns with best practices for reproductive health.

## Introduction

Emergency contraception is a reliable means of preventing undesired pregnancies after unprotected sexual intercourse, contraceptive failure, or rape incidents if used within a specified time frame [1]. According to the World Health Organization, emergency contraceptives (ECs) can prevent up to 95% of potential pregnancies [2]. Functioning primarily by impeding or delaying ovulation, the modalities of emergency contraception involve hormonal methods, selective progesterone receptor modulators, and intrauterine device (IUD) insertion [3]. Among these, the most frequently used method is hormonal ECs, typically comprised a combined oral contraceptive containing ethinyloestradiol and levonorgestrel or high-dose progestogen-only pills. These ECs are intended to be used within 72 hours of unprotected intercourse [4].

ECs are known by various terms such as "second chance," "post-coital contraception," and "morning-after pill," and in Tanzania, they are commonly referred to as "P2 pills." ECs are not intended as substitutes for regular contraception according to the manufacturer's guidelines and the American College of Obstetricians and Gynecologists [5]. Manufacturers of ECs advocate alternative contraceptive methods if there is a need for frequent contraception within a short duration, as there is limited research data regarding repetitive use [5]. It should be noted that consistent usage of ECs may expose the user to possible adverse effects such as menstrual disturbances [5]. Other reported side effects include nausea and headaches, with some patients reporting different short-term adverse reactions such as breast tenderness, abdominal pain, dizziness, and fatigue. Combined ECs are not recommended for individuals with active migraine because of the elevated risk of ischemic stroke [6,7]. It is also crucial to note that ECs do not protect against sexually transmitted diseases (STDs), including HIV. Therefore, other contraceptive methods, such as internal or external condoms, should be considered [6].

Recent studies have shown that 57% of young women and 48% of young men are sexually active by the age of 18 years [7]. In 2016, the Tanzania Demographic Health Survey ranked the country with the highest adolescent fertility rate (17th). This survey also revealed an increase in the adolescent fertility rate from 116 to 132 births per 1,000 girls aged 15-19 between the 2010 and 2015/2016 periods. Furthermore, it was reported that by the age of 15-19 years, 22% of young women would have experienced their first pregnancy, contributing to a 4% rise in teenage pregnancy since 2010. Consequently, by 2016, one in four adolescents aged 15-19 had begun childbearing, indicating that a substantial proportion of these pregnancies progress to full term, resulting in the birth of an infant, despite a general decline in fertility trends [8]. Moreover, long-term use of ECs has been associated with contraceptive failure, subsequently increasing the risk of undesired pregnancies [9,10]. Research conducted in various countries has revealed a lack of knowledge regarding ECs. Unfortunately, this issue has not been adequately documented in Tanzania [10-12]. The primary aim of this study was to analyze the consumption trends of ECs over six consecutive years and provide insights into consumption trends, including exploring the reasons behind the observed fluctuations in consumption. We investigated trends in EC utilization between 2018 and 2023 using data from the Tanzania Medicines and Medical Devices Authority (TMDA) importation database. The findings of this study established a baseline consumption trend and determined the future demand for ECs, including suggesting immediate interventions. 

## Materials and methods

Research design

This was a retrospective, longitudinal, analytical study to evaluate EC consumption trends. The study used importation data on human medicines from January 1, 2018 to December 31, 2023 and stored in the TMDA Regulatory Information Management System (RIMS) database.

Study setting

This study was conducted in the United Republic of Tanzania, which currently has a total population of 61,741,120, of which 47.3% (n=14,992,288) were women of childbearing age. Among them, 24% (n=4,722,158) were teenage girls aged 13-19. Tanzania is bordered by eight countries and the Indian Ocean to the East. Tanzania is an entry point for pharmaceutical imports through seaports and airports. Key entry points include the Dar es Salaam Airport, Sea Harbor, Kilimanjaro Airport, and terrestrial border checkpoints in Sirari, Horohoro, Namanga, Tunduma, and Mutukula.

Data collection method

Data were sourced from the importation records of oral ECs, as provided by the TMDA, functioning as the National Medicines Regulatory Authority, which oversees the importation of medicines into mainland markets. Importers must obtain an importation permit through a stringent evaluation process, after which permits are recorded in TMDA's RIMS. These records, drawn from the TMDA database of imported medicines, were utilized to approximate the utilization of ECs across the country, with the underlying assumption that all documented imports are consumed within Tanzania under the regulatory oversight of the TMDA. The study excluded records with missing permit numbers, reference numbers, or permit issue dates due to the inability to accurately determine the year of importation. Records outside the designated timeframe were omitted.

Data collection and analysis

Data on imported medicines, including date, generic and brand names, strength, quantity, company (suppliers or importers), price, currency, product manufacturer, and country of origin, were gathered from the TMDA database [13]. First, we amalgamated the source data files and transposed the consolidated data using Microsoft Power BI software (Microsoft® Corp., Redmond, WA). The strength, pack size, and quantity of ECs were converted to milligrams and grams for further quantification. We visualized trends in EC usage using a series of tables and graphs.

Computation of medication volumes adhering to the formula

$\text{Volume} = \text{quantity} \times \text{pack size}$ We defined levonorgestrel's defined daily dose (DDD) as 0.75 mg to standardize dosage. The total number of additional units was calculated using the following formula:

$\text{DDD}_{\text{total}} = \frac{\text{volume} \times \text{strength in mg}}{\text{DDD}}$

Then, the calculated DDD per 1000 inhabitants per year (DID) was computed as

$\text{DID} = \left(\frac{\text{DDD}_{\text{total}}}{\text{population}}\right) \times \frac{1000}{365}$

This allowed for the estimation of annual drug consumption per 1000 individuals, adjusted for daily intake, and provided a standardized measure of drug utilization across different populations.

IBM SPSS Statistics for Windows, Version 26 (Released 2019; IBM Corp., Armonk, New York, United States) facilitated the input of annual utilization data, enabling us to conduct time series and regression analyses to ascertain the yearly consumption trends of ECs.

We employed rigorous time-series analysis to model and forecast the DID for ECs in Tanzania. Utilizing the time-series model, we extended our predictive analysis between December 2023 and 2026 to provide a comprehensive view of the future consumption trends of ECs in the region. This analysis incorporated advanced modeling techniques, including autoregressive integrated moving average (ARIMA) and exponential smoothing models. Expert modeler functionality and optimal model selection were also used after evaluating the unique characteristics of the data, such as seasonality and trend components. Additionally, we applied curve-fitting techniques to enhance the model's accuracy in capturing the underlying patterns of EC usage over time and reported a 95% confidence interval.

## Results

There were 114 EC importation records between 2018 and 2023, of which 95.6% (n=109) were oral ECs and 4.4% (n=5) were IUDs. Within the period of study, the contribution of imported volumes of ECs increased from 1.9% in 2018 to 60.1% in 2023 (Table 1).

**Table 1:** Annual frequency of importation permits, volumes, and contribution of the imported volumes of emergency contraceptives

Year	Number of consignments	Volume of consignments	% volume of consignments
2018	13	31,178,520	1.9
2019	9	13,398,000	0.8
2020	9	18,738,000	1.1
2021	14	222,077,862	13.4
2022	25	373,255,304	22.6
2023	44	992,735,200	60.1
Total	114	1,651,382,886	100

**Figure 1:**
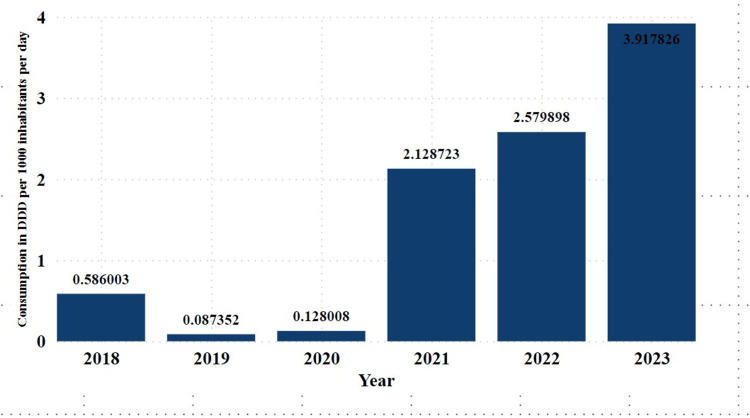
Annual consumption trends of ECs expressed by DDD per 1000 inhabitants per day in Tanzania from 2018 to 2023 ECs: emergency contraceptives; DDD: defined daily dose

In the studied timeframe, the year 2023 had the highest DID at 3.917826. This remarkable increase was 4,983.06% higher than that in 2019 when the lowest DID of 0.0873552 was recorded. Significantly, the year 2023 comprised 41.63% of the total DID across the six years. Consumption decreased in 2019 and 2020, followed by a rapid increase from 2021 to 2023 (Figure 1). The reduction in EC consumption from 2019 to 2020 was 36.9% compared with that in 2021 and 2022.

**Figure 2:**
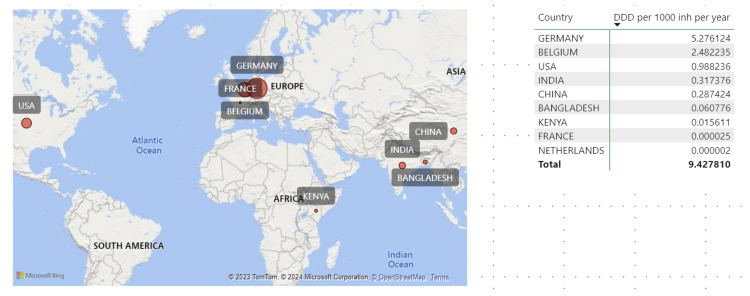
Countries supplying ECs to Tanzania expressed by DDD per 1000 inhabitants per day between 2018 and 2023. The size of the bubble in the map corresponds to the quantity of DIDs ECs: emergency contraceptives; DDD: defined daily dose; DID: DDD per 1000 inhabitants per day The figure was created using Microsoft Power BI Desktop software.

The leading countries that imported ECs were Germany, Belgium, and the USA, whereas the Netherlands, France, and Kenya imported the lowest amounts of ECs. Germany and Belgium contributed about 82.9%, the USA and India contributed about 13.7%, and the remaining countries accounted for 3.4% of all contraceptives imported into Tanzania between 2018 and 2023 (Figure 2).

**Figure 3:**
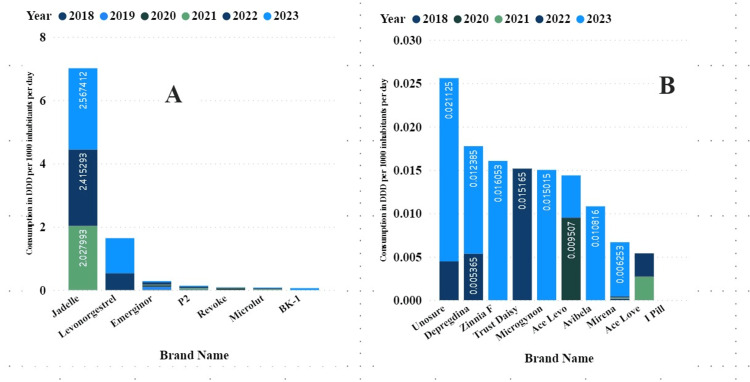
Brands of ECs consumed in Tanzania expressed by DDD per 1000 inhabitants per day between 2018 and 2023. Panel A shows products with a total DDI above 0.005 and panel B shows those with totals above 0.005 ECs: emergency contraceptives; DDD: defined daily dose; DID: DDD per 1000 inhabitants per day

The partner and private sectors contributed 94% and 6% of all DIDs of utilized contraceptives. Different brands of oral ECs were imported during the study period, with P2 being the most well-known brand, followed by Emerginor among the users. In 2023, there was an increase in new brands imported into Tanzania because of high imports compared to the other study years (Figure 3).

**Figure 4:**
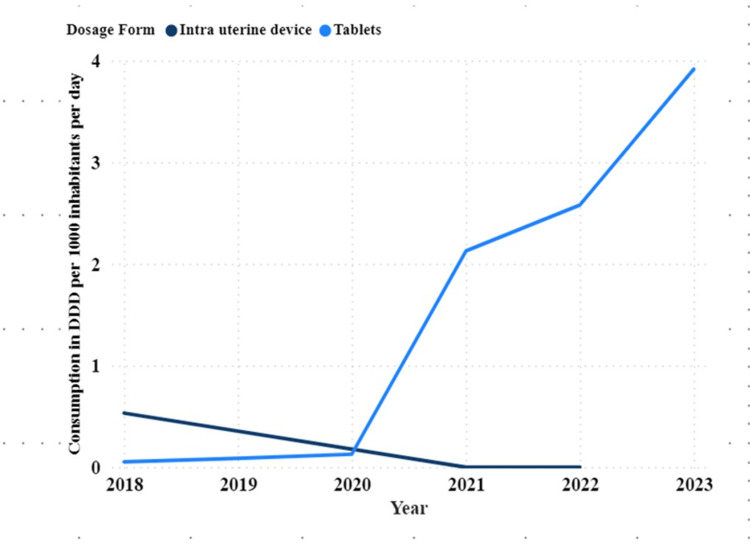
Trends of dosage forms of ECs consumed in Tanzania expressed by DDD per 1000 inhabitants per day (DID) in Tanzania from 2018 to 2023 ECs: emergency contraceptives; DDD: defined daily dose; DID: DDD per 1000 inhabitants per day

Based on the administration route, there was a high proportion of oral contraceptive use (87.5%) compared to IUD use (12.5%). Oral ECs constituted 95.7% compared to IUDs 4.38% (Figure 4).

**Figure 5:**
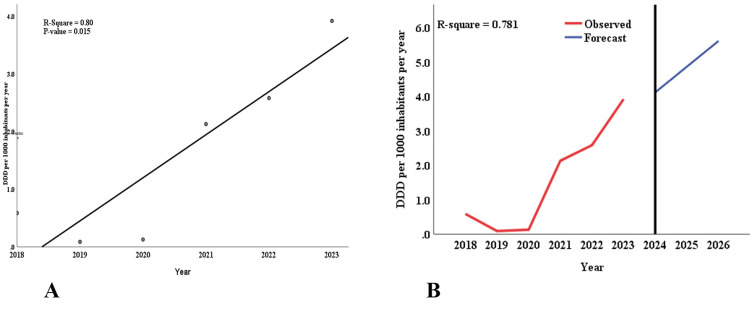
Trends in total consumed ECs from 2018 to 2023. The linear curve estimation for (A) the overall EC consumption shows an increasing trend. (B) The Holt model with autoregressive integrated moving average (ARIMA) shows an upward forecast until 2026 ECs: emergency contraceptives; DDD: defined daily dose

The linear analysis from 2018 to 2023 showed an upward trend in utilizing ECs in Tanzania, with an R-square value of 0.80 and a p-value of 0.015 (Figure 5A). Time-series modeling further highlighted this trend, projecting the DID from 2018 to 2026. This model achieved an R-squared value of 0.781, indicating that it could explain approximately 78.1% of the variation in DID. The observed data demonstrate an increase in DID from 0.586003 in 2018 to 3.917826 in 2023 (Figure 5B). The forecasts suggest a continuing upward trend, with the DID expected to grow from 4.114157 in 2024 to 5.607726 in 2026 (Figure 5B).

## Discussion

This is the first pharmacoepidemiological study to investigate the EC consumption patterns in Tanzania. It typically uses a non-experimental design based on secondary data from regulatory practices. The study shows that alarming trends of consumption increase from 2021 to 2023. However, we cannot ascertain whether this increase is due to the increased access to ECs over the counter. This is likely due to the extensive spread of accredited drug dispensing outlets and other pharmaceutical outlets nationwide. Its predominance in consumption affirms the reduction in condom utilization and increased risk of sexually transmitted infections (STIs), including HIV/AIDS [14] and the greater likelihood of unwanted/unplanned pregnancies due to inappropriate use. 

We could not ascertain region-specific trends, but they were generalized for the whole country and, thus, are likely to mask regions with populations at increased risk. The study falls short in ascertaining pharmacoepidemiological risk factors for the hazards of the continual misuse of ECs owing to a lack of precise level data. The estimates were based on distributed and accessed contraceptive items. The exclusion of incomplete records of importation and distribution may occlude actual contraceptive units that could have meaningful interpretations. However, these were considered non-response items because this is more or less nationwide data with monopolized regulations; hence, the 11% non-response rate is acceptable.

The increase in the importation of ECs from 2021 to 2023, with a decrease in annual imports from 2019 to 2020, could have been caused by the health service disruptions during the coronavirus epidemic. This calls for efforts to advocate the effective utilization of reproductive and sexual health services among youths [15]. An increase in the utilization of ECs indicates an increase in knowledge and reduced negative social attitudes toward the use of ECs. Since most consumers of ECs are youths between 16 and 25 years old, an increase in the utilization of ECs suggests reduced unwanted and early pregnancies among schoolgirls, as suggested by a cross-sectional study [14]. Interestingly, this finding is inconsistent with the corresponding decrease in fertility among the general population of women of childbearing age.

There has been a general decline in fertility in Tanzania over the past decade, from 5.4% children in 2010 to 4.8% children in 2022 [8,12], while 22% of adolescent girls have ever been pregnant by 15-19 years. The proportion of contraceptive use among sexually active adolescents is unreported, as the Demographic and Health Survey Management Information System (TDHS-MIS) only measures the use of contraceptives among married women, which tends to underestimate their use at the national level [8]. The TDHS-MIS also reported that 45% of sexually active unmarried women of unspecified age used contraceptives (36% using modern methods and 8% using traditional methods), which still conflicts with the actual trends. The ever-pregnancy estimate of adolescents by 19 years of age is a good indicator of the non-use of contraceptives of any kind among this young group. This argument underscores the importance of immediate interventional measures, as adolescent girls and young women present 80% of new HIV infections in this era of high antiretroviral therapy coverage at all levels. 

Most studies examining the acceptability of ECs by potential users and health providers have demonstrated acceptance of this method. ECs are generally perceived as particularly useful for adolescents who initiate sexual activity without contraceptive protection and in cases of rape or accidents during the use of other contraceptives [11,16]. According to different studies, IUDs are preferred by women who have given birth [17].

The observed upward trend in utilizing ECs in Tanzania, as delineated by linear and time series analyses, suggests a significant shift in reproductive health practices among the population. The steady increase in DID from 2018 to 2023 and the projected continuation of this trend until 2026 highlight the growing acceptance and use of ECs and raise questions about the underlying factors driving this trend. This trend could reflect changes in societal attitudes, increased awareness, and accessibility of ECs, or a potential paradigm shift in sexual health education and practices. The continuation of this trend underlines the importance of ongoing monitoring and evaluation of contraceptive use patterns to inform public health strategies and to ensure the availability and appropriate use of ECs. Further research into the social, economic, and policy factors influencing these trends will be pivotal for shaping effective health interventions and ensuring that the use of ECs aligns with best practices for reproductive health.

A multifaceted approach involving improved access and education across all age groups is essential to enhance the use of IUDs and counteract the biases against this contraceptive method. Facilitating open knowledge and offering free access to services can also significantly promote using IUDs, potentially reducing reliance on ECs and their associated adverse effects. Concurrently, revising importation policies to support the influx of ECs by decreasing import taxes and consequently increasing retail prices can foster increased use and consumption. The availability and affordability of contraceptives in health centers and pharmacies hinges on these importation trends and pricing strategies. Finally, along with the promotion of IUDs and ECs, condom use should be advocated to mitigate the increasing incidence of STDs and STIs, particularly among adolescents, thereby addressing a comprehensive spectrum of reproductive health and preventive measures.

Study limitations

While shedding light on the surging usage of ECs for over six years, this study has inherent limitations. Its retrospective approach primarily draws on importation data, which may not fully represent the diverse motivations behind EC usage or capture the entirety of user experiences. Additionally, the analysis did not incorporate user-specific data, such as direct feedback or qualitative insights, into personal contraceptive choices, potentially overlooking significant behavioral, and social factors influencing EC consumption. These limitations call for future research endeavors to integrate longitudinal and qualitative methodologies to better understand the EC utilization patterns and their broader implications.

## Conclusions

The observed increase in EC consumption over the past six years, paralleled by an increase in adolescent fertility rates, suggests prevalent misuse and misinformation regarding ECs. This scenario signifies an urgent need for targeted educational interventions and awareness programs. Such efforts should aim to disseminate accurate information on the proper use of ECs and encourage planned contraceptive practices among key demographics, particularly among sexually active adolescents and young women who are also at a higher risk of HIV infection. Educating these groups on ECs and the importance of condoms as a preventive measure against STDs and unplanned pregnancies is critical.

Given these circumstances, stakeholders, including health ministries and regulatory bodies, are called upon to leverage these data for policy formulation and execution of educational strategies to optimize the distribution and utilization of contraceptives. There is a pressing need to address the intertwined challenges of increasing EC consumption, teenage pregnancies, and HIV incidence using a multifaceted approach. In addition, future research should delve into user experiences and practices related to EC usage to inform the development of interventions that can mitigate the long-term health effects of ECs. Establishing cohorts for such studies could provide valuable insights into the health outcomes associated with prolonged EC use and guide more effective public health strategies and contraceptive use policies.
